# Activity of Alkaloids on Peptic Ulcer: What’s New?

**DOI:** 10.3390/molecules20010929

**Published:** 2015-01-08

**Authors:** Raphaela Francelino do Nascimento, Igor Rafael Praxedes de Sales, Rodrigo de Oliveira Formiga, José Maria Barbosa-Filho, Marianna Vieira Sobral, Josean Fechine Tavares, Margareth de Fátima Formiga Melo Diniz, Leônia Maria Batista

**Affiliations:** Department of Pharmaceutical Sciences, Federal University of Paraiba, João Pessoa 58051-970, PB, Brazil; E-Mails: raphaelafrancelino@gmail.com (R.F.N.); igor_caraubas@hotmail.com (I.R.P.S.); rodrigo.formiga@hotmail.com (R.O.F.); jbarbosa@ltf.ufpb.br (J.M.B.-F.); mariannavbs@gmail.com (M.V.S.); josean@ltf.ufpb.br (J.F.T.); margareth@ltf.ufpb.br (M.F.F.M.D.)

**Keywords:** alkaloids, gastroprotective, peptic ulcer, natural products, review

## Abstract

Peptic ulcer is a common disease characterized by lesions that affect the mucosa of the esophagus, stomach and/or duodenum, and may extend into the muscular layer of the mucosa. Natural products have played an important role in the process of development and discovery of new drugs, due to their wide structural diversity and present, mostly specific and selective biological activities. Among natural products the alkaloids, biologically active secondary metabolites, that can be found in plants, animals or microorganisms stand out. The alkaloids are compounds consisting of a basic nitrogen atom that may or may not be part of a heterocyclic ring. This review will describe 15 alkaloids with antiulcer activity in animal models and *in vitro* studies.

## 1. Introduction

Peptic ulcer disease (PUD) is an illness characterized by lesions in the gastrointestinal mucosa that may penetrate the muscularis layer. Stomach lesions are located preferentially along the small curvature in the transition zone between the body and the antrum, whereas in the duodenum those lesions are located in the bulb or duodenal bulb [1]. PUD affects approximately 10% of the population worldwide [2] and represents not only costs to the individual, but also for public health [3]. Both gastric and duodenal ulcers, affect men more than women, being the most prevalent the duodenal one in younger people, while gastric ulcer is more prevalent in the elderly [4].

The development of peptic ulcer is complex and involves a multifactorial process, which occurs by an imbalance between aggressive and defensive factors of the gastric mucosa [5]. The gastric mucosa is continuously exposed to harmful substances and factors, whether endogenous as acid secretion, peptic activity and biliary secretion or exogenous as the presence of *Helicobacter pylori*, the use excessive of alcohol, tobacco and anti-inflammatory non-steroidal (NSAIDs), besides stressful life habits [5,6].

However, despite this assault, under normal conditions the gastric mucosa is able to resist damage from 0.1 mol/L HCl and pepsin present in the gastric lumen by a variety of defense mechanisms which include as a first line the defense pre-epithelial component, mucus-bicarbonate-phospholipid; then an epithelial component, the continuous layer of epithelial cells connected by tight gap junctions, which generates bicarbonate, mucus, phospholipids, trefoil peptides, prostaglandins, and heat shock proteins; In addition, there are other mechanisms such as cell renewal; continuous blood flow; production of nitric oxide and hydrogen sulfide; and sensory innervation [7].

Natural products are evolutionarily designed and chemically differentiated from the majority of synthesized molecules. Besides, they are capable of regulating biological systems because they are able to interact with various macromolecules [8]. Thus, natural products have played an important role in the process of development and discovery of new drugs [9], due to their wide structural diversity and because they present mostly specific and selective biological activities [10].

Among the natural products the alkaloids, biologically active secondary metabolites, that can be found in plants, animals or microorganisms, stand out. Biosynthetically, the alkaloids are derived from amino acid biosynthesis or transamination processes, and they are classified according to the amino acid that yields the nitrogen atom as well as the part of its skeleton for the synthesis of the alkaloid in question [11]. Thus, the alkaloids are compounds consisting of a basic nitrogen atom that may or may not be part of a heterocyclic ring [12].

Alkaloids are endowed with diverse biological activities, being already used in therapy as pharmacological tools. Among the reported biological effects, they present antitumor [13,14], anticholinergic [15], diuretic [16], sympathomimetic [17], antiviral [18], antihypertensive [19], hypnoanalgesic [20], antidepressant [21], myorelaxant [22], antimicrobial [23], antiemetic [24] and antiinflammatory properties [25]. However, there are also reports of toxic effects to humans [13], thus, it is necessary the use of different experimental models to understand the exact mechanism of the molecules under study, in order to have the real knowledge of their effect.

In the course of our continuing search for bioactive natural plant products, we have published reviews on crude plant extracts and plant-derived compounds with potential medicinal uses [25,26,27,28,29,30,31,32,33,34,35,36,37,38], including alkaloids. In a previous paper we have presented a review on plants of the American continent with antiulcer activity [39], and the gastroprotective, duodenal and peptic antiulcer activity of flavonoids [40], tannins [41] and alkaloids [42].

In this article, we review studies published between January 2008 and September 2014 on alkaloids with peptic ulcer activity. The survey was conducted in databases such as SciFinder Scholar^®^, Science Direct^®^ and PubMed^®^ using as keywords: gastroprotective or antiulcer or antiulcerogenic activity and alkaloids. The alkaloids cited in this review were selected according to pharmacological action demonstrated in specific experimental models to evaluate the anti-ulcer activity and/or through studies in order to discover their mechanism of action.

## 2. Results and Discussion

### 2.1. Rutaecarpine

**Figure 1:**
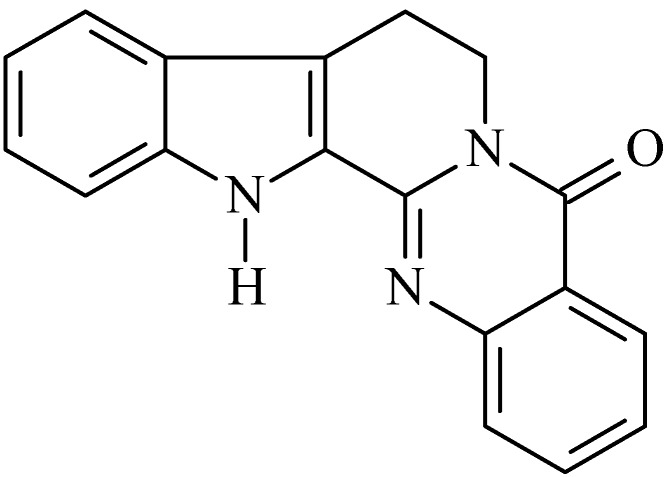
Rutaecarpine.

The alkaloid rutaecarpine (8,13-dihydroindolo-[2′,3′:3,4]-pyrido[2,1-b]quinazolin-5(7*H*)-one, Figure 1) is an indolopyridoquinazoline alkaloid [43], first isolated by Asahina and Kashiwaki from a ketone extract of *Evodia rutaecarpa* [44]. Since then, rutaecarpine has been found in other genera of the family *Rutaceae*, as *Evodia*, *Horit*, *Zanthoxylum*, *Phellodendron* among the others. Dried fruit of *Evodia rutaecarpa* (called Wu-Chu-Yu) is used for treating disorders of the gastrointestinal tract and dysentery in traditional oriental medicine [45].

Intravenous administration of rutaecarpine in rats at doses of 100 or 300 mg/kg significantly reduced the ulcer index and pH value when compared to the control group in ulcer models induced by both acetylsalicylic acid (ASA) and stress. ASA promotes back-diffusion of H^+^ the mucosa and signicantly increased the ulcer index, indicting the development of hemorrhagic and non-hemorrhagic lesions of the gastric mucosa [46].

Under stress sympathetic nervous system (SNS) and parasympathetic (SNP) stimulation occurs. Sympathetic stimulation promotes arteriolar vasoconstriction, causing reduction in blood flow in mucosa, promoting local hypoxia and ischemia, generating ROS, lipid peroxidation and depletion of glutathione levels. The stimulation of SNP increases with consequent worsening motility of the gastrointestinal muscle contraction, mucosal ischemia leading to increased secretion of acid [47,48].

Calcitonin gene-related peptide (CGRP) is a neuropeptide predominantly found in capsaicin-sensitive sensory neurons, which are in abundance in the gastrointestinal tract. These nerves are involved in gastroprotection, CGRP being a potential mediator of this process, the synthesis of this neurotransmitter is regulated by transient receptor potential vanilloid subfamily member 1 (TRPV1). The beneficial effects of CGRP include increased mucosal blood flow, inhibition of gastric acid secretion, prevention of apoptosis and oxidative injury [49].

Wang *et al.* [46] demonstrated that the gastroprotective effect by rutaecarpine (300 or 600 µg/kg, p.o.) in models of ASA or stress-induced gastric ulcer was decreased by the pretreatment with capsaicin (50 µg/kg, p.o.) an antagonist of the TRPV1. According to studies by Aizawa *et al.* 2001 [50], *N*^G^-nitro-L-arginine methyl ester (L-NAME), inhibitor of nitric oxide synthase (NOS), reduces the levels of CGRP. Endogenously, NOS is inhibited by L-arginine analogue asymmetric dimethylarginine (ADMA) formed by hydrolysis of proteins in which the arginine residues are methylated from arginine methyltransferase, while being degraded by dimethylarginine dimethylaminohydrolase (DDAH) [51]. Thus, in the ethanol-induced ulcer model in rats, ruteacarpine increases the activity of DDAH reducing the levels of ADMA, enhancing NO synthesis and reducing the gastric damage [51]. Rutaercapine also present gastroprotective effects by increasing the release of CGRP by two mechanisms: TRPV1 activation and increased activity of DDHA.

### 2.2. Phenylquinoline

**Figure 2:**
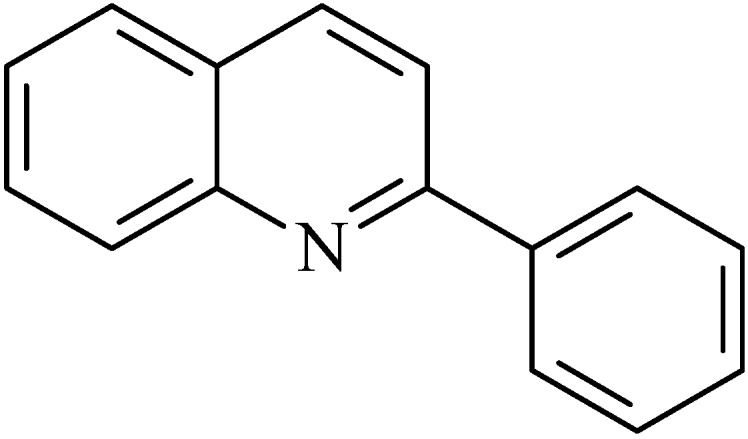
2-Phenylquinoline.

2-Phenylquinoline (Figure 2) is an alkaloid that has been obtained from the bark of *Galipea longiflora* Krause [52,53,54].

Zanatta *et al.* [52] evaluated the gastroprotective effect of 2-phenylquinoline obtained from the bark of *G. longiflora* starting from the total alkaloids fraction that was then chromatographed on a column of silica gel (0.063–0.20 nm, 100 g) and eluted with ethyl acetate in *n*-hexane to obtain 2-phenylquinoline [52].

Pharmacological studies using the HCl/etanol-induced ulcer model displayed the anti-ulcer activity of 2-phenylquinoline at the dose of 50 mg/kg. In this model, ethanol promotes destabilization of mucus-bicarbonate-phospholipid layer, leading to retrodiffusion of H^+^ ions, with consequent damage to epithelial cells and mast cell activation inducing the release of inflammatory mediators, that allow the migration of neutrophils to the injured area, releasing reactive oxygen species that elevate the cellular damage and result in tissular necrotic damages. The 2-phenylquinoline inhibited ulcerative lesions in 60.2% ± 7.9%, being this result significant when compared with the negative control [52,55].

Additionally, when 2-phenylquinoline is evaluated in the pylorus ligation model, it is possible to observe changes in the gastric juice parameters, like a reduction in the volume of gastric juice and the total acidity, while gastric pH compared was increased significantly compared with the control group [52].

In addition, the involvement of nitric oxide and gastric mucus from the models used to determine contents of mucus adhered to gastric mucosa and ulcer model induced by ethanol with pre-treatment L-NAME it was observed [52].

Mucus is the first line of defense of the mucous membrane, being secreted by epithelial cells and consisting of 95% water and 5% mucin, a polymerized glycoprotein that forms a gel. This secretion is stimulated by gastrointestinal hormones such as gastrin and secretin, prostaglandin (PGE_2_) and cholinergic agents [6].

Nitric oxide, on the other hand, is formed from the conversion of L-arginine to citrulline by the action of nitric oxide synthase (NOS). The NO is implicated in the increase of mucus secretion, bicarbonate and blood flow in the gastric mucosa, as well as the inhibition of gastric acid secretion, providing gastroprotection and eliminating free radicals with consequent reduction of gastric lipid peroxidation [56,57,58]. After administration of L-NAME an increase of 42% in mucus production was observed and, there was a reduction in the inhibition of 53.2% to 48.4%, suggesting that at least in part, nitric oxide participates in the gastroprotection [52].

### 2.3. Nicotine

**Figure 3:**
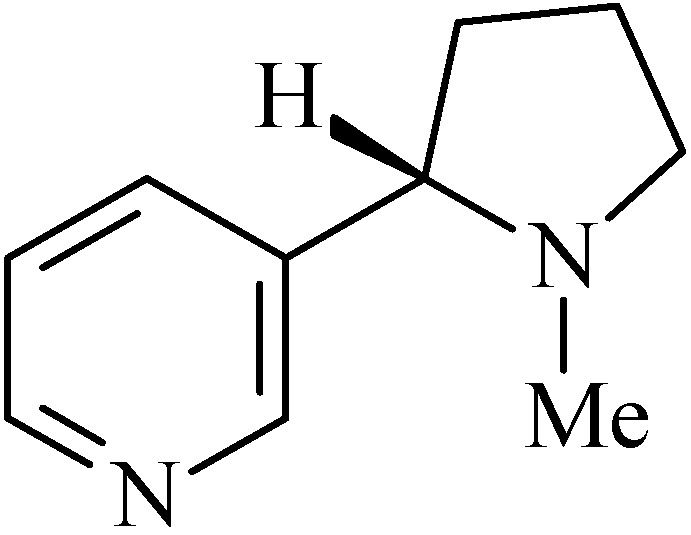
Nicotine.

Nicotine (3-(1-methylpyrrolidin-2-yl)pyridine, Figure 3) is an alkaloid commonly found in plants of Solanaceae family, obtained especially from the dried tobacco leaves of *Nicotiana tabacum* L., and the presence of this alkaloid in human matrices happens first by exposure to tobacco smoke [42,59].

Nicotine causes psychological and physical dependence when used chronically. This substance interacts with cholinergic receptors in adrenal medulla, autonomic ganglia, neuromuscular junction and brain of mammals [60]

In the ASA-induced ulcer model, nicotine reduced the ulcerative lesions, probably due to an increase of the mucus secretion as reflected by the increase in pH and intragastric volume. However the nicotine did not increase the mucosal blood flow, but rather decreased it by about 18% [60].

Meanwhile, in another study animals were pretreated with nicotine (0.05 mg/mL in tap water) for 28 days and after treated with ethanol. There was an increase in gastric mucosal injury in the animals that received the pretreatment with nicotine, while the nicotine itself had no effect on gastric lesions. Besides, in ethanol-induced gastric mucosal injury an increase of L-arginine analogue asymmetric dimethylarginine (ADMA) levels was observed in gastric juice and the animals that received the chronic pretreatment with nicotine had an increased level of ADMA [61].

*In vitro* studies were also conducted, where mucosal epithelial cells were treated with nicotine (3 or 10 µmol/L) in the presence or absence of ethanol, and then the average concentration of ADMA in culture and the proportion of cells undergoing apoptosis were measured. Much higher levels of ADMA as well as increased apoptosis were observed in cells treated with nicotine and ethanol. Therefore, nicotine increases the levels of ADMA and reduced NO levels, leading to the damage caused by ethanol to the gastric mucosa [61].

### 2.4. Rohitukine

**Figure 4:**
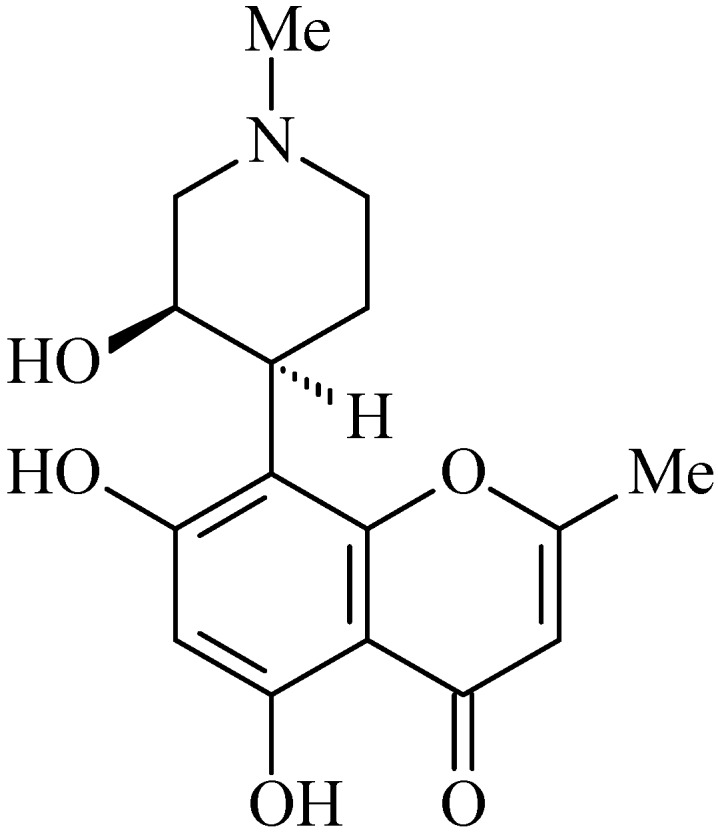
Rohitukine.

Rohitukine (5,7-dihydroxy-8-((3*S*,4*R*)-3-hydroxy-1-methylpiperidin-4-yl)-2-methyl-4*H*-chromen-4-one, Figure 4) is a chromane alkaloid which was originally isolated from plants of the Meliaceae family—*Amoora rohikuta* and later *Dysoxylum binectariferum* [62,63,64].

Rohitukine presents several biological activities such as anti-inflammatory, immunomodulatory, anti-cancer [62], contraceptive [65], anti-leishmania [66] and gastroprotective effects [67].

To evaluate the anti-ulcer activity rohitukine several protocols were performed in an ulcer model induced by stress, where animals were treated with the alkaloid in doses of 10, 20 and 40 mg/kg orally; in the ulcer model induced by pylorus ligature, where rohitukine was tested at a dose of 20 mg/kg, with the objective of evaluating the gastric juice, peptic activity and levels of mucin; and the model of ulcer induced by ethanol. Moreover, gastrin levels, intracellular calcium ions and the activity of the H^+^/K^+^ ATPase were measured, being these two last tested *in vitro* [67].

Rohitukine decreased the index of ulcerative lesion compared to the control group in the stress-induced ulcer model as in the ethanol model. This alkaloid reduced levels of gastric juice and free/total pepsin, blocked the activity of H^+^/K^+^ ATPase, normalized gastrin levels and reduced levels of Ca^2+^ in parietal cells, furthermore, it enhanced gastric mucosa defense mechanisms by increasing the levels of prostaglandin E_2_ (PGE_2_) and mucin [67].

The parietal cells are responsible for the production of gastric secretion [47]. This secretion creates a favorable medium for the conversion of pepsinogen to pepsin (an important enzyme in the protein digestion process), facilitating the absorption of iron, calcium and vitamin B_12_, as well as preventing bacterial overgrowth and possible infections [68].

Moreover, the parietal cells contain a small number of intracellular canaliculi and a series of tubular vesicles that have the pump H^+^/K^+^ ATPase. The stimulation of these cells leads to fusion of the tubule vesicles with the membrane of the apical cell, and the activation of H^+^/K^+^ ATPase, that starts to pump H^+^ into the gastric lumen and K^+^ into the intracellular medium. The K^+^ in turn returns into the gastric lumen by co-transport with Cl^−^ ions in the apical portion of the cell [47,69].

The primary stimulation of acid secretion in the gastric mucosa is performed by histamine, gastrin and acetylcholine [47]. Histamine is released from cells similar to enterochromaffin (ECL) and stimulates the parietal cell directly via H_2_ receptors coupled to protein G_s_. Gastrin is released from the G cells and stimulates parietal cells either directly or indirectly by interacting with receptors of cholecystokinin-2 (CCK-2) present in ECL cells, inducing the release of histamine [47,69]. The acetylcholine released from postganglionic neurons, stimulate the parietal cell receptors directly or indirectly via M_3_, M_2_ and M_4_ receptors coupled to the inhibition of secretion of somatostatin [47,69,70].

Somatostatin released by D cells interacts with sstR_2_ receptors, whereas PGE_2_ acts on the EP_3_ receptor, both located on parietal cells and coupled to G_i_ protein, and then they inhibit the production of adenosine monophosphate (cAMP), leading to reduction of acid secretion [47,69].

### 2.5. Methoxycanthin-6-one

**Figure 5:**
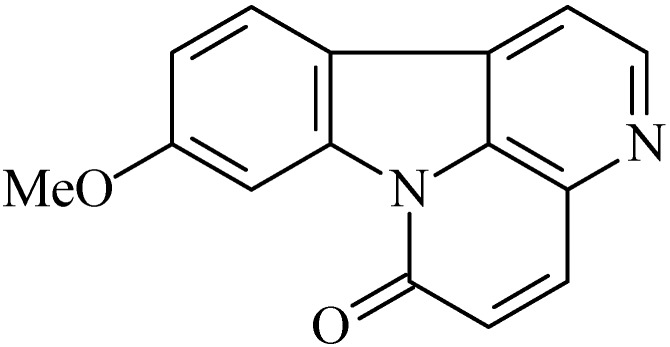
2-Methoxycanthin-6-one.

The alkaloid 2-methoxycanthin-6-one (9-methoxy-6*H*-indolo[3,2,1-de][1,5]naphthyridin-6-one, Figure 5) was isolated from the crude methanol extract of the stem wood of *Quassia amara* L. [71]. This alkaloid demonstrated potent actives related to male antifertility in male rats [72] and antiulcer activity [73].

To evaluate the gastroprotective activity of 2-methoxycanthin-6-one, the ulcer model induced by indomethacin was realized in doses of 12.5, 25 and 50 mg/kg, p.o. and 1, 2 and 4 mg/kg, i.p. [73]. Indomethacin is a nonselective cyclooxygenase inhibitor that impairs the synthesis of prostaglandins. As a result of the inhibition of PGE_2_ and PGI_2_ synthesis there is a decrease in gastric mucus and bicarbonate production and changes in blood flow, thus, reducing the protective barrier of the normal mucosa and also the ability to repair mucosal cells [74]. The results shown that 2-methoxycanthin-6-one produced a dose-dependent gastroprotective effect on indomethacin-induced gastric lesions in rats [73].

Moreover, the pylorus ligature model was used wherein was performed using the dose which produced better effect 4 mg/kg i.p in ulcer model induced by indomethacin. In this model, cimetidine, a histaminergic antagonist, was used, in order to determine whether the mechanism of action of 2-methoxycanthin-6-one has any participation of the histaminergic pathway. 2 Methoxycanthin-6-one decreased gastric secretion, however, in the presence of cimetidine, this effect was reduced, demonstrating that is likely an alkaloid H_2_ antagonist [73].

### 2.6. Chelerythrine

**Figure 6:**
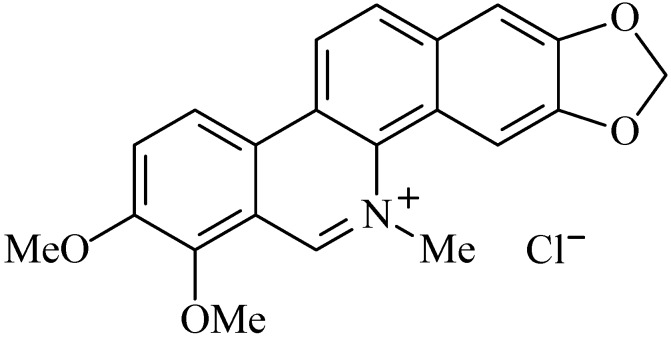
Chelerythrine chloride.

Chelerythrine chloride (1,2-dimethoxy-N-methyl(1,3)benzodioxolo(5,6-c)phenanthridinium chloride, (Figure 6) is an alkaloid commonly found in the Papaveraceae and Rutaceae families which can be classified as a quaternary benzo[c]phenanthridine alkaloid [75]. Among its reported biological activities, there are anti-inflammatory effects [76] and interaction with certain proteins such as protein kinase C (PKC) and mitogen-activated protein kinase phosphatase-1 [77].

Li *et al.* [75] evaluated the gastroprotective effect of chelerythrine against ethanol-induced gastric mucosal lesions. A pre-treatment with chelerythrine at doses of 1, 5 and 10 mg/kg, p.o. was realized in mice for four days, followed by administration of absolute ethanol (0.2 mL/animal). A decrease of the ulcerative level in a dose dependent manner was observed when compared to the control group [75]. Gastric juice acidity and mucus production were also evaluated. Animal groups pre-treated with this alkaloid showed a significant increase of pH when compared with the control group.

From the histopathological studies it was observed that pre-treatment with chelerythrine resulted in a reduction in ulcer area, and reduction or absence of submucosal edema and leucocytes infiltration. Furthermore, a reduction in the overproduction of mucus and NO in the serum and gastric tissues was observed [75]. This overproduction of NO occurs during innate immune system responses when activated macrophages produce NO to play its bactericidal activity [78].

Besides, by immunohistochemistry analysis and western blot it was possible to observe an effect of chelerythrine on protein expression of NF-κB p65 in gastric tissue and the effect of chelerythrine on cytokines was determined by ELISA [75].

Cytokines such as tumor necrosis factor-alpha (TNF-α) and interleukin-6 (IL-6) and IL-10 play an important role in acute inflammation and in the maintenance and regulation of gastric mucosal damage [79,80]. TNF-α has the capacity to induce fever, stimulates the production of acute-phase proteins by the liver and causes endothelial cell activation [55], while IL-6 causes high levels of active lymphocytes, neutrophils and monocytes/macrophages in the inflamed area [75].

The expression of these pro-inflammatory mediators may be increased by the activation of NF-κB subunits (p65 and p50) and can be reduced by the inactivation of NF-κB whereby PKC phosphorylates the subunit 65 [81,82]. Chelerythrine reduced the levels of TNF-α and IL-6, pro-inflammatory cytokines, in serum and tissue of rats exposed to ethanol as well as reduced levels of p65 subunit of NF-κB, showing the initial stages of inflammation action [75].

### 2.7. Piplartine

**Figure 7:**
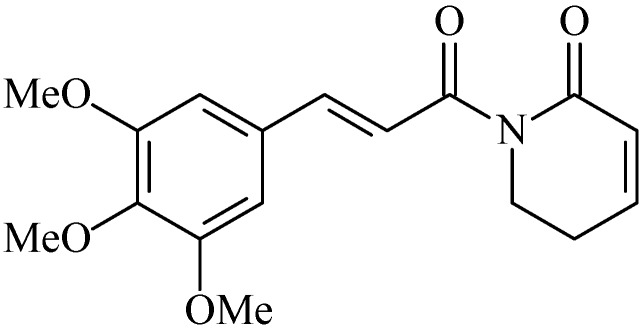
Piplartine.

Piplartine, (piperlongumine, 5,6-dihydro-1-[(2*E*)-1-oxo-3-(3,4,5-trimethoxyphenyl)-2-propenyl]-2(1*H*)-pyridinone, Figure 7) is the major alkaloid of *Piper longum* L. (long pepper). It is found in species of the Piperaceae family [83].

Piplartine has great economic and medicinal importance [83]. Several pharmacological activities are reported for piplartine such as cytotoxic [84], genotoxic [85], antitumor [86], antiangiogenic [86], antinociceptive [87], anxiolytic [88], antidepressant [88], anti-atherosclerotic [89], antidiabetic [90], antibacterial [91], leishmanicidal [92], trypanocidal [93], schistosomicidal [94], and gastroprotective properties [95].

To evaluate the gastroprotective activity of this alkaloid, initially the gastric ulcer model induced by ethanol was performed in which pirplatine was tested at doses of 4.5 mg/kg, p.o. Additionally, the gastric acid secretion from the pylorus ligation was evaluated, where pirplatine was tested at doses of 4.5 and 15 mg/kg i.d. This evaluation was undertaken to determine the gastric mucous, the levels of reduced glutathione and evaluate the activity of the H^+^/K^+^ ATPase pump [95].

Piplartine reduced the rate of ulcerative lesions compared to the control group, and did not affect the levels of mucus, however, it increased the levels of GSH, reduced the activity of H^+^/K^+^ATPase pump, reduced the volume and total acidity of the gastric juice and reversed the stimulatory effect of pentagastrin (CCK_2_ receptor agonist) on gastric acid secretion, demonstrating that piplartine is a CCK_2_ receptor antagonist [95].

The gastric mucosa is constantly assaulted by various endogenous and exogenous factors [96], however it has several defense mechanisms, such as the mucus layer that acts as a physical barrier [6] and reduces the damage caused by oxidative stress [97], reduced glutathione, that acts directly in the oxidizing system [98]. Piplastine has gastroprotective effects by increasing levels of GSH and its anti-secretory effect [95].

### 2.8. N-Isopropylmethylanthranilate

**Figure 8:**
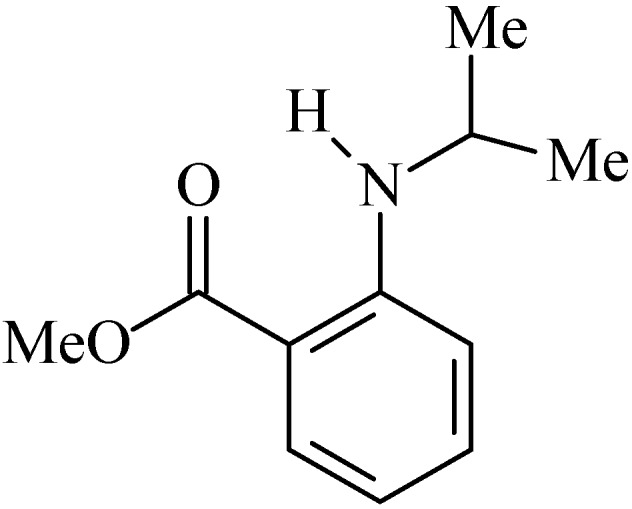
*N*-Isopropylmethylanthranilate.

*N*-isopropylmethylanthranilate (NIM, Figure 8), was initially identified in the essential oil of *Choisya ternata* Kunth (Rutaceae) by the gas chromatography and gas chromatography-mass spectrometry techniques, albeit in small amounts, then e NIM was synthesized in order to obtain sufficient amounts to test its possible biological activities [99].

NIM is a volatile alkaloid and due to this property, it were tested in animal models as a potential anxiolytic, antidepressant and antinociceptive drug [99,100]. After these studies, Radulović and colleagues [101] hypothesized that due to the antinociceptive activity observed in this alkaloid it could have anti-inflammatory activity and consequently have anti-ulcer effects since peptic ulcer is an inflammatory phenomenon.

Initially, Radulovićet *et al.* [101] evaluated the effects of NIM (200 mg/kg, p.o.) on the integrity of the gastric mucosa to verify a possible ulcerogenic effect of these substances. However the alkaloid did not alter the architecture of the gastric tissue. After that, the gastroprotective activity of NIM (50, 100 and 200 mg/kg, p.o.) in animal models of ulcers induced by NSAIDs (diclofenac 80 mg/kg, p.o.) and ethanol was investigated. All evaluated doses (*p* < 0.05) of the two substances protected the stomach from diclofenac and ethanol-induced lesions [101].

### 2.9. N-Methylmethylanthranilate

**Figure 9:**
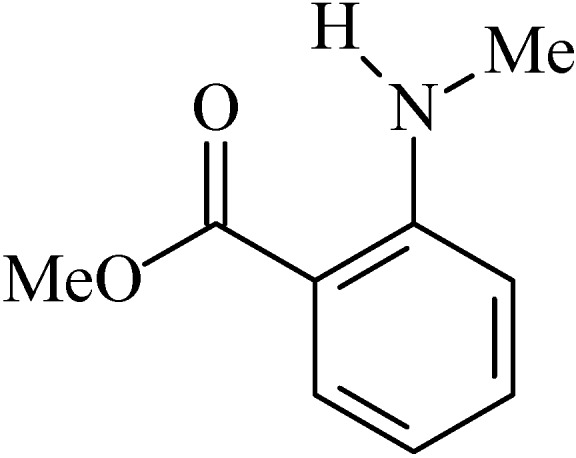
*N*-Methylmethylanthranilate.

The alkaloid *N*-methylmethylanthranilate (NMM, Figure 9) is considered an important marker of the essential oil of *Citrus recutita*, found in species of the Rutaceae family, particularly in the Rutoideae and Aurantioideae subfamiles [99]. In the *Choisya ternata* Kunth (Rutaceae) species this alkaloid was found in small quantities by chromatographic techniques, however, it is readily synthesized, and it can thus be obtained in sufficient quantities to test its pharmacological effects [99].

The NMM has demonstrated antinoceptive action in a dose-dependent manner [99], as well as a significant anxiolytic and antidepressive effect [100]. Moreover, it showed gastrorprotective effects in models of ulcer induced by ethanol and diclofenac in doses of 50, 100 and 200 mg/kg, po, when compared to the control group [101].

### 2.10. (+)-O-Methylarmepavine

**Figure 10:**
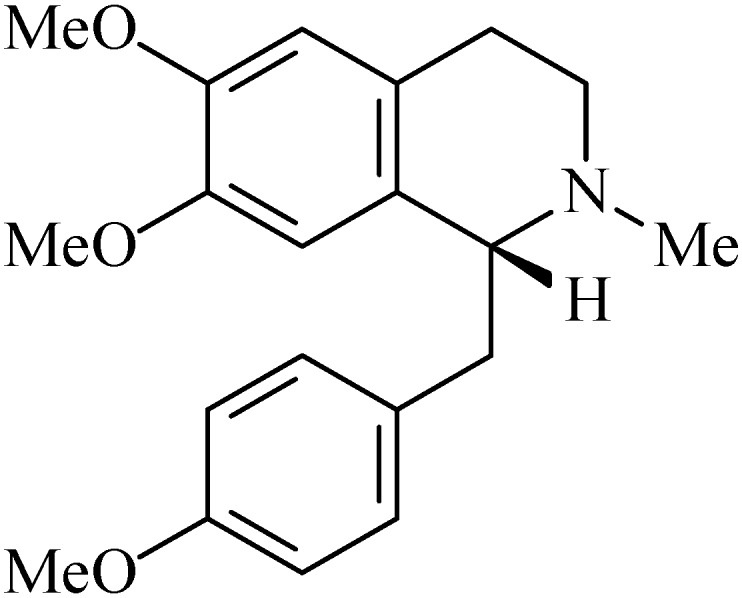
(+)-*O*-Methylarmepavine.

(+)-*O*-Methylarmepavine (6,7-dimethoxy-1-[(4-methoxyphenyl)methyl]-2-methyl-1,2,3,4-tetra-hydroisoquinoline, Figure 10) is a benzylisoquinolinic alkaloid isolated from the chloroform fraction of the twigs of *Annona squamosa* (Annonaceae), popularly known as sugar apple [102,103] and also isolated from the bark and root of *Xylopia parviflora* [104] This alkaloid was also first described in the genus Papaver [105].

Vila-Nova and collaborators [106] recently showed that (+)-*O*-methylarmepavine (100, 50, 25, 12.5, and 6.25 µg/mL) had leishmanicidal activity against both the promastigote and amastigote forms of *Leishmania chagasi* in a diphenyltetrazolium (MTT) assay. (+)-*O*-Methylarmepavine (treatment during 14 days—0.3, 1.0 and 3.0 mg/kg, p.o.) promoted stimulation of the immune system in BALB/c mice and the best results were seen for the higher dose [107].

In another pharmacological assay the alkaloid (10–100 µg/mL) was tested on H^+^/K^+^-ATPase isolated from gastric microsomes of rat. (+)-*O*-Methylarmepavine inhibited gastric H^+^/K^+^-ATPase activity in comparison with control group with a percentage inhibition of 53.84 (IC_50_: 111.83 µg/mL) [99]. (+)-*O*-Methylarmepavine (20 mg, p.o.) reduced the plasma gastrin levels in ethanol-induced gastric ulcer to 102.8 ± 6.6 pg/mL compared to the control (127.5 ± 3.7 pg/mL, *p* > 0.05). In the same study, this alkaloid did not stimulate the production of prostaglandin E2 (PGE_2_) [108].

### 2.11. N-Methylcorydaldine

**Figure 11:**
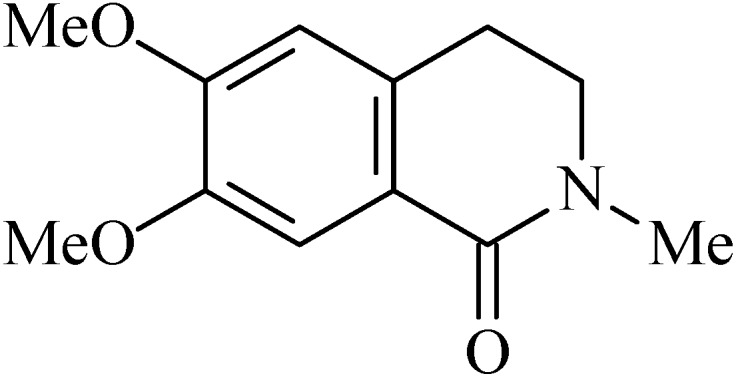
N-Methylcorydaldine.

*N*-Methylcorydaldine (6,7-dimethoxy-2-methyl-3,4-dihydroisoquinolin-1-one, Figure 11) is an isoquinolinic alkaloid. The first report of isolation of this alkaloid from *Papaver bracteatum* (Papaveraceae) was made by Theuns and collaborators [109]. It is also found in aerial parts of *Hammada articulata* (Chenopodiaceae) [110], twigs of *Annona squamosa* (Annonaceae) [102], *Fumaria vaillantii* (Fumariaceae) [111] and twigs of *Hernandia ovigerous* L. (Hernandiaceae) [112].

Pharmacological tests performed with N-methylcorydaldine have shown that the alkaloid (10–100 µg/mL) inhibits gastric H+/K+-ATPase activity in comparison with control, with a percentage inhibition of 71.43 (IC_50_: 60.9 µg/mL). N-Methylcorydaldine (20 mg, p.o.) reduced the plasma gastrin level in ethanol-induced gastric ulcer to 96.8 ± 8.9 pg/mL (*p* < 0.05) but did not stimulate the production of prostaglandin E_2_ (PGE_2_) [108].

### 2.12. Isocorydine

**Figure 12:**
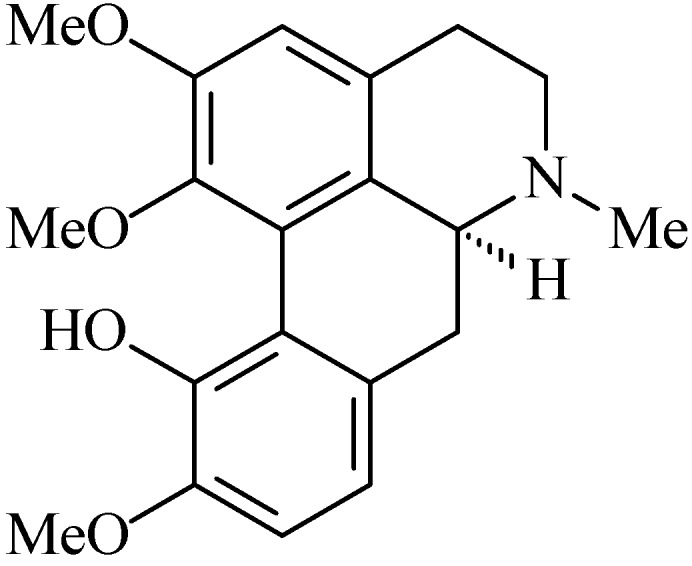
Isocorydine.

Isocorydine (6a*S*)-1,2,10-trimethoxy-6-methyl-5,6,6a,7-tetrahydro-4*H*-dibenzo[de,g]quinoline-11-ol, Figure 12) is an aporphine benzylisoquinolinic alkaloid. This alkaloid has been isolated from many plant parts and species such as: twigs of *Annona squamosa* (Annonaceae) [102], roots and rhizomes of *Isopyrum thalictroides* (Ranunculaceae) [113], roots of *Dactylicapnos scandens* (Papaveraceae) [114], *Corydalis saxicola* (Papaveraceae) [115], *Doryphora sassafras* (Monimiaceae) [116], leaves of *Aquilaria sinensis* (Thymelaeaceae) [117], stems and leaves of *Stephania cephalantha* (Menispermaceae) [118] and *Aconitum brachypodum* (Ranunculaceae) [119].

The first pharmacological study of this alkaloid was reported by Chen, Zhang and Wang [120] who demonstrated its spasmolytic activity on the biliary system of guinea pigs or rabbits. Isocorydine also exhibited cytotoxicity on different human hepatocellular carcinoma (HCC) cell lines by mechanisms that involve stopping the cell cycle and induction of apoptosis [121,122]. Isocorydine showed weak gastric H^+^/K^+^-ATPase activity in comparison with control a with percentage inhibition of 35.46% (IC_50_: 88.42 µg/mL). Isocorydine (20 mg/kg p.o.) did not alter the plasma gastrin level when compared to the control 112.0 ± 10.2 pg/mL (*p* > 0.05) and did not stimulate the production of PGE_2_ [108].

### 2.13. Canthin-6-one

**Figure 13:**
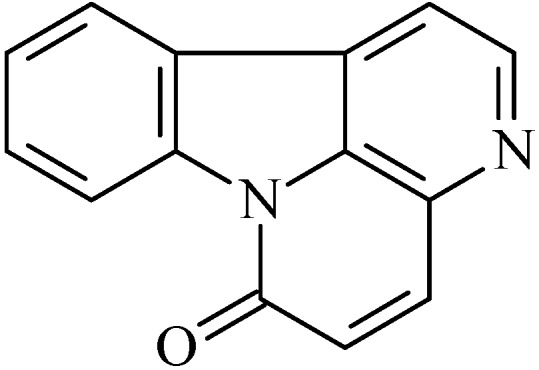
Cathin-6-one.

Canthin-6-one (6*H*-indolo[3,2,1-de][1,5]naphthyridin-6-one, Figure 13) is an indole alkaloid isolated from the rizhomes of *Simaba ferruginea* A. St.-Hil. (Simaroubaceae) [109]. The antifungal activity of this compound as investigated in *Saccharomyces cerevisiae* as a possible modulator positive effect of alkyl chain desaturase enzyme systems [110]. This compound also has gastroprotective activity [111].

A study was conducted by Almeida and colleagues [109] to investigate the mechanism involved in the gastroprotective activity of canthin-6-one (20 mg/kg, p.o.). The results suggest that the alkaloid has gastroprotective effects (against ethanol and indomethacin-induced ulcer) by stimulating nitric oxide production, acting by modulating the levels of pro-inflammatory cytokines (IL-8) and decreasing the activity of myeloperoxidase (MPO) and lipid peroxidation, suggesting a possible antioxidant activity.

### 2.14. Peganine and Derivatives

**Figure 14:**
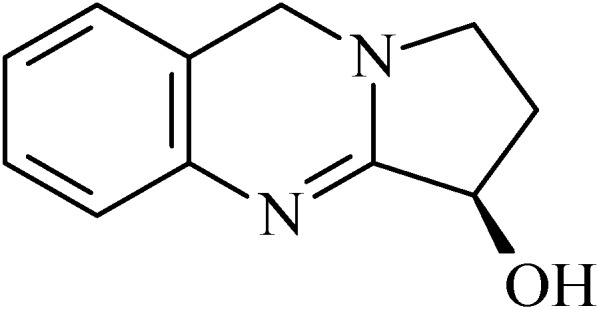
Peganine.

Peganine (known as vasicine, 1,2,3,9-tetrahydropyrrolo[2,1-b]quinazolin-3-ol, Figure 14) is a natural alkaloid found in *Adhatoda vasica* (Acanthaceae) [123,124] and *Peganum harmala* (Zygophyllaceae) [125]. Based on the structure of peganine (PEG) two alkaloids: vasicinone hydrochloride (VAS, 2,3-dihydro-3-hydroxypyrrolo(2,1-b)quinazolin-9(1*H*)-one hydrochloride) and acetyl vasicinone (AVA) [125] were synthesized in the laboratory

PEG is a substance with antioxidant activity (*in vitro* DPPH assay) and antibacterial activity (agar diffusion assay). It also has cytotoxic activity toward different strains of cancer, with better results seen in tests performed with Sp2/O-Ag14 cells (IC_50_ = 50 mg/mL to >100 mg/mL) [123,126].

The three substances PEG (20 and 40 mg/kg, p.o.), VAS (20 amg/kg, p.o.) and AVA (20 mg/kg, p.o.) showed gastroprotective activity on cold restraint-induced gastric ulcer models in rats. VAS and AVA showed poor protection (37.5%) then compared with PEG (50.0 and 58.5 for 20 and 40 mg/kg, respectively) [125].

Peganine (20 mg/kg, p.o.) also protected the stomach from the ulcerative lesions induced by ethanol (89.41% of protection), non-steroidal anti-inflammatory drugs (NSAIDs) (58.50%) and pylorus ligature (62.50%). The mechanism of action of the gastroprotective activity of PEG involves the reduction of gastric acid secretion that was verified by biochemical parameters of gastric juice (*in vivo*) and inhibitory activity of the proton pump (*in vitro*). Furthermore PEG acted by modulating the levels of PGE_2_ and displayed antioxidant activity an *in vitro* DPPH assay [125].

### 2.15. 4-Guanidinobutyric Acid

**Figure 15:**
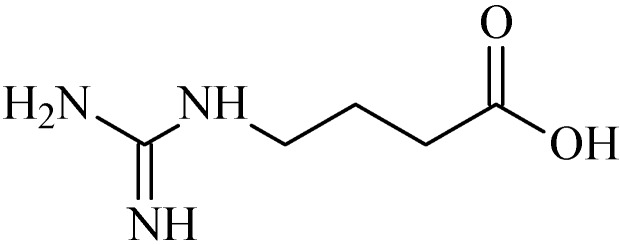
4–Guanidinobutyric acid.

4-Guanidinobutyric acid (4GBA, Figure 15) is an alkaloid present in herbal medicines, mammalian brain, fish and shellfish [127], besides being included in the guanidino compound family [128].

Hwang and Jeong [128] bserved in *in vitro* studies that 4GBA was able to inhibit the growth of *H. pylori* at a dose of 100 mM. Furthermore, 4GBA inhibited approximately 70.8% of gastric lesions induced by HCl/ethanol at a dose of 100 mg/kg, higher than cimetidine (150 mg/kg), the positive control (approximately 40.9% inhibition). 4GBA reduced indomethacin-induced gastric lesions in rats (38.8% inhibition) and slightly decreased the gastric secretion volume (5.7 ± 1.45 mL) compared to the control (6.4 ± 2.99 mL) [128]. 4GBA also exhibited cytotoxicity in SNU638 cells (IC_50_ = 43.7) and AGS cells (IC_50_ = 75.1 µM). These results suggest that 4GBA inhibits gastric cancer cell growth [128].

## 3. Conclusions

In summary, this review demonstrates the great importance of stimulating studies with natural products, especially alkaloids, for the discovery of new therapeutic alternatives in healing of peptic ulcer due to these metabolites’ broad and differentiated mechanisms of action, thus potentially benefiting the population in general by promoting access to medicines and offering a better quality of life.
